# Use of Testosterone‐Lowering Medication in Patients With Intellectual Disabilities and Problematic Sexual Behaviour: A Case Series

**DOI:** 10.1155/crps/6191292

**Published:** 2026-05-31

**Authors:** Jacob Rong Bentsen, Torbjørn Lien Kjær, Magnus Roland Balleby, Jimmi Nielsen

**Affiliations:** ^1^ Glostrup Copenhagen University Hospital, Glostrup, Denmark, gentoftehospital.dk; ^2^ Aarhus Universitets Hospital, Aarhus, Central Denmark Region, Denmark, auh.dk

**Keywords:** case report, cyproterone acetate, hypersexuality, intellectual disability, leuprorelin, paraphilic disorder, testosterone-lowering medication

## Abstract

**Background:**

Testosterone‐lowering medication (TLM) is used in severe cases of paraphilic disorders and problematic sexual behaviour, but its use in patients with intellectual disability is not well characterised.

**Method:**

A nationwide survey identified five male patients with intellectual disability who had received TLM in specialised outpatient clinics.

**Results:**

Four patients showed reduced problematic sexual behaviour during treatment; three relapsed after discontinuation. Adverse effects included osteoporosis, hypertension, depression and suppressed sexual development. Monitoring and access varied regionally.

**Conclusions:**

TLM may be effective in selected patients with intellectual disability but requires careful monitoring and ethical oversight.

## 1. Introduction

First‐line treatment for paraphilic disorders and problematic sexual behaviour typically consists of counselling, psychotherapy—most commonly cognitive–behavioural approaches—and selective serotonin reuptake inhibitors (SSRIs). In individuals with intellectual disabilities, psychotherapeutic interventions often require adaptation to cognitive level and communication abilities and are usually delivered within structured behavioural programmes. In more severe cases or when there is a significant risk of harm, testosterone‐lowering medication (TLM) may be considered as part of a multimodal treatment approach [1–3]. TLM includes medroxyprogesterone acetate (MPA), cyproterone acetate (CPA) and gonadotropin‐releasing hormone (GnRH) analogues such as goserelin, triptorelin and leuprorelin [3]. MPA decreases GnRH and luteinising hormone (LH) levels through negative feedback on the hypothalamic–pituitary–testicular axis and thereby decreases testosterone production [4]. Like MPA, CPA is a steroidal antiandrogen with progestogenic properties, in addition, CPA acts as an androgen receptor antagonist [4]. GnRH is a hypothalamic peptide hormone secreted in pulses to stimulate pituitary release of LH and, to a lesser extent, FSH. GnRH analogues initially activate GnRH receptors, but sustained stimulation leads to receptor downregulation, reduced LH and FSH secretion, and consequently, decreased testosterone production [5].

TLM is typically initiated during prison trials, psychiatric special measures, or municipal placement but may also be voluntarily sought by motivated patients. Intellectual disability is a wide spectrum of reduced cognitive performance with onset before the age of 18 and is associated with somatic comorbidities, for example, epilepsy and dementia and psychiatric comorbidities [6]. Problematic sexual behaviour can be a challenge for patients with intellectual disability and has led to long‐term institutionalisation in closed institutions [7, 8]. Patients with intellectual disabilities may benefit less from psychotherapeutic interventions, which leaves a treatment gap for this patient group where TLM might have a role [3, 8].

TLM for paraphilia is ethically complex and historically controversial.

Motivation is often linked to easing sanctions, raising concerns about the validity of voluntary consent. For patients with cognitive impairments, informed consent is further challenged due to limited understanding of efficacy and side effects. In this paper, cognitive impairment is defined according to the ICD‐10 classification. In cases of severe intellectual disability, where IQ is in the range of 34 or below and communicative abilities are severely compromised, consent must be given by next of kin or legal guardian [7].

The aim of this case series is to display the diversity of use of TLM in patients with intellectual disability treated voluntarily at the five specialised outpatient regional clinics for intellectual disability in Denmark.

## 2. Method

In autumn 2023 and spring 2024, we surveyed the five regional specialised outpatient clinics for intellectual disability in Denmark to identify patients treated with TLM. Consultant psychiatrists were contacted directly and asked whether they had any patients who were currently receiving, or had previously received, treatment with CPA or GnRH analogues.

Clinics provided data on current and past TLM use, including duration, estimated effect on problematic sexual behaviour and aggression and side effects. Additional data included age, gender, residence type, occupation, pedagogical support, degree of intellectual disability, paraphilias, other problematic sexual behaviours, psychiatric and somatic comorbidity, BMI, blood pressure, recreational drug use, legal sanctions and use of psychotropic and somatic medication. Information was also collected on TLM effects on testosterone, FSH, LH, haemoglobin, alanine transferase, HbA1c, electrocardiogram and bone densitometry. Written informed consent was obtained from all patients or their legal guardians to participate in this case series. According to national regulations, this type of case series, based solely on anonymised clinical data and without intervention, did not require prior approval from a Human Research Ethics Committee.

## 3. Results

The survey identified five cases from two regions; four were currently receiving TLM. Cases are summarised below and in Table 1.

**Table 1:** An overview of the five included cases.

Patient	Diagnosis	Other pharmacological treatment	TLM treatment	Outcome	TLM side effects
1	Severe intellectual disability, excessive sexual drive and disorder of sexual preference	Zuclopenthixol 30 mg valproate 600 mg Previously: quetiapine, olanzapine, risperidone, haloperidol and levomepromazine without sufficient effect	Unknown dose and duration of treatment CPA (ended in 1982)	No effect on behaviour	Depression
67 years			Sex hormone levels were not measured		No information regarding bone densitometry
2	Moderate to severe intellectual disability, autistic disorder, non‐organic psychosis, disorder of sexual preference and paedophilia and pyromania	Clozapine 300 mg, valproate 600 mg, diazepam 10 mg PRN, pregabalin 150 mg, chlorprothixene 50 mg and 50 mg PRN enalapril, bisacodyl, magnesia and potassium	Duration 15 years Inj. CPA 300 mg/10 days Goserelin 10.8 mg/5 weeks	Reduction of problematic sexual behaviour, but not sufficiently Problematic sexual behaviour occurred during treatment	Osteopenia Hypertension Obstipation
34 years			P‐TEST 0.25–0.38 nM P‐FSH, 1.0 nM P‐LH, 0.2–0.3 nM		
3	Intellectual disability, disorder of sexual preference, reactive attachment disorder in childhood and ADHD	Sertraline 100 mg, atomoxetine 120 mg Previously: Lisdexamfetamin, atomoxetine, methylphenidate, risperidone	Duration 2 years (15 −17 years) Leuprorelin 11.25 mg/11 week	Sufficient reduction of problematic sexual behaviour Recurrence of problematic sexual behaviour after treatment was terminated	Reduced development of male sex characteristics
20 years			P‐TEST, Decreased from 16.6 to 3.32 nM		No information regarding bone densitometry
4	Intellectual disability, ADHD, harmful use of alcohol, epilepsy and disorder of sexual preference	Escitalopram 20 mg, clonazepam 1 mg, valproic acid 100 mg and diazepam 10 mg PRN	Duration several years Inj. CPA 400 mg/2 weeks	Reduction of problematic sexual behaviour but worsening after termination of treatment	The patient could not cooperate to bone densitometry
54 year			P‐TEST, 5.3 nM		
5	Intellectual disability and disorder of sexual preference	Alendronate 70 mg/weekly metformin 200 mg, dapagliflozin 10 mg, ramipril 5 mg, atorvastatin 80 mg Daily inhalations of Anoro ellipta	Duration > 20 years Leuprorelin 22.5 mg/12 week, CPA 300 mg/2 weeks	Reduction of problematic sexual behaviour but worsening after termination of treatment	Osteoporosis Spine fracture
64 year			P‐TEST, 0.24 nM P‐SHBG, 95 nM LH is 0.3 nM		

Abbreviations: CPA, cyproterone acetate; LH, luteinizing hormone; P‐TEST, plasma testosterone; SHBG, sex hormone binding globulin.

### 3.1. Case 1

A 67‐years‐old male diagnosed with severe intellectual disability accompanied by motor stereotypies, grimaces and no verbal language. He exhibited diminished impulse control, hypersexual and voyeuristic behaviour increasing in periods of distress. The patient had a low functioning level and was easily frustrated, leading to aggression and self‐injury. He showed no psychotic symptoms, substance abuse, somatic illness or history of criminal sanctions. In 1982, he was treated with CPA with questionable effect and developed depressive symptoms. Goserelin was considered, but consent from the legal guardian was not obtained. Treatment with citalopram, zuclopenthixol and valproate had no effect on problematic sexual behaviour and a limited effect on aggression.

### 3.2. Case 2

A 34‐year‐old male with moderate to severe intellectual disability, autism spectrum disorder, hypersexuality, paedophilic disorder, and pyromania reported auditory hallucinations. He exhibited limited verbal language, reduced empathy, impaired social cognition and poor impulse control. Strict behavioural supervision was required to prevent public masturbation and inappropriate conduct. He had a conviction under special criminal legislation and resided in a secure institution for 9 years. Since age 19, he had received antipsychotics, CPA, and goserelin. TLM reduced inappropriate sexual behaviour, although the index offence occurred during treatment. Adjunctive clozapine further reduced sexual and violent behaviour. He now lives in a specialised open residential facility with staff support. Following relocation, aggressive and sexually inappropriate behaviour increased.

### 3.3. Case 3

A 20‐year‐old male diagnosed with mild intellectual disability, hypersexuality, unspecified paraphilic disorder, childhood reactive attachment disorder and ADHD. He exhibited excessive masturbation and paedophilic behaviour. There was no history of psychotic symptoms, self‐injury, somatic illness or substance use. At age 15, leuprorelin acetate was initiated due to problematic sexual behaviour, resulting in reduced masturbation and inappropriate conduct. Treatment was discontinued after 2 years due to suppressed development of male secondary sex characteristics. After discontinuation, problematic sexual behaviour remitted. Psychostimulants had no significant effect on impulse control. Before leuprorelin, risperidone had been prescribed but discontinued due to coagulation disturbances and gingival bleeding. He is currently treated with sertraline and atomoxetine without improvement in sexual behaviour. He resides in a 1:1 staffed residential facility, participates in day employment. He has been charged with sexual offences. Reevaluation for reinstating leuprorelin treatment is underway.

### 3.4. Case 4

A 54‐year‐old male diagnosed with mild intellectual disability, ADHD, harmful use of alcohol and epilepsy. He presented with hypersexuality and violent sexual fantasies and had multiple arrests for inappropriate physical contact with women. He had also assaulted and threatened coresidents, demanding they undress. He was treated with disulphiram, which effectively reduced alcohol use. He resided in a supported living facility and worked as a cleaner at a local pub. Treatment with escitalopram and CPA reduced problematic sexual behaviour. After several years, CPA was discontinued at the patient’s request, and inappropriate behaviour recurred. CPA was reinitiated in depot form with good effect.

### 3.5. Case 5

A 64‐year‐old male with mild intellectual disability had been institutionalised since childhood. From adolescence, he exhibited hypersexuality and impaired impulse control, resulting in multiple attempted rapes and indecent assaults. He also displayed violent behaviour toward staff and had multiple convictions leading to placement in closed institutions. Treatment with leuprorelin and CPA was initiated 35 years ago during institutionalisation. Sexual preoccupation, including fantasies, pornography use and masturbation, ceased. However, treatment was discontinued after 8 months due to emerging aggression, suspected to be related to TLM. Concurrent use of recreational drugs may have contributed. Following discontinuation, sexual preoccupation returned, and he attempted sexual assault on a staff member during escorted leave. He subsequently consented to resumed treatment with leuprorelin and CPA, which has continued for over 20 years with sustained adherence. Since reinitiating TLM, no further sexually aggressive behaviour has occurred, and he has lived in an open institution. He developed osteoporosis attributed to TLM and was treated with alendronate. He sustained a spinal fracture and underwent routine bone densitometry. Due to the severity and risk of past offences, TLM has not been discontinued.

## 4. Discussion

This case series describes five patients who received TLM for problematic sexual behaviour. Four patients showed a reduction in problematic sexual behaviour, leading to increased autonomy in daily life. Previous studies summarised in the World Federation of Societies of Biological Psychiatry (WFSBP) guidelines indicate that TLM (including CPA, MPA and GnRH agonists) is associated with substantial reductions in sexual drive and paraphilic behaviour, with response rates around 80%–90% reported in several antiandrogen studies. Clinical response in these studies was primarily defined by reductions in self‐reported sexual fantasies, masturbation frequency and clinician‐rated paraphilic behaviour and in some cases, supported by biological suppression of testosterone levels. However, outcomes were often short‐term, based largely on open‐label or small cross‐over designs, and robust long‐term controlled data using standardised behavioural or recidivism endpoints remain limited [3]. Three patients experienced recurrence of paraphilic behaviour after discontinuation of TLM, supporting its therapeutic effect. This is consistent with previous findings, although prior studies have not focused exclusively on individuals with intellectual disability [9–11].

The cases included individuals with mild to severe intellectual disability according to ICD‐10, none of whom were considered suitable for psychotherapy. All resided in institutional settings with professional staff and presented at least a moderate risk of sexual violence. SSRIs were used in only two patients, possibly due to concerns about behavioural activation, including self‐injury and aggression, which are particularly relevant in this population [12], and consequently treatment with antipsychotics was prioritised. Four out of five patients received antipsychotic medication at some point during the treatment trajectory. Antipsychotics were prescribed to manage comorbid agitation, psychotic symptoms or behavioural dysregulation, which is in line with general practice within intellectual disability [13]. While not indicated for the core treatment of paraphilic disorders, antipsychotics may modulate sexual behaviour. The WFSBP 2020 guidelines for the pharmacological treatment of paraphilic disorders acknowledge the use of antipsychotics when psychiatric comorbidity is present [3, 7].

Four patients discontinued treatment due to side effects: depression, increased aggression, or concerns regarding prolonged pubertal suppression resulting in suppressed development of male secondary sex characteristics. Patients 2 and 5 developed osteopenia or osteoporosis, a known adverse effect [3]. One patient declined bone densitometry; data were unavailable for two others. One patient developed hypertension.

Combination therapy with CPA and a GnRH analogue was required in patients 2 and 5, consistent with WFSBP recommendations for cases unresponsive to monotherapy. Plasma testosterone was regularly monitored in all patients except one, showing marked reductions during treatment. Patients receiving combination therapy achieved castration‐level testosterone (<1.7 nM), while those on monotherapy with CPA or a GnRH analogue reached hypogonadal levels (~5 nM). These outcomes align with WFSBP pharmacodynamic data: GnRH analogues induce pituitary downregulation with testosterone suppression <1.7 nM, whereas CPA exerts a dual antiandrogenic and progestogenic effect with variable hormonal outcomes (2–8 nmol/L) [3]. Both patients treated with combination therapy had histories of multiple sexual assaults; patient 2 had previously been placed in a maximum‐security facility.

Reduced aggression occurred in three of the patients, which is in line with previous findings [10, 11, 14, 15]. Patient 5 reported increased aggression during initial TLM, though concurrent use of recreational drugs may have contributed. Notably, aggression did not reoccur following reinitiating TLM.

Limitations of this case series include the absence of standardised rating scales for assessing problematic sexual behaviour. Assessments were based on observations by staff and healthcare professionals. Side effects and paraclinical investigations were inconsistently monitored due to limited patient cooperation. These factors may have biased efficacy and safety estimates and limit comparability with other studies but reflect the challenges of guideline implementation in this population. Varying levels of social support may also have influenced the risk of problematic sexual behaviour[8].

Additional patients may have received TLM, but the lack of a national register limits oversight. Few cases reduce clinical experience, possibly affecting monitoring and prescribing. Findings support the need for centralised, specialised national treatment centres.

Despite evidence supporting the effectiveness of TLM for paraphilic disorders, the treatment remains controversial [16]. In three of five Danish regions, no TLM cases were reported despite specialised clinics. The reason for regional variation remains unclear. Similar variability was seen in Germany, linked to forensic history [17, 18].

In some cases, consent was given by a guardian, raising ethical concerns. Such dilemmas may deter psychiatrists from initiating TLM.

In conclusion, TLM appears effective in reducing problematic sexual behaviour in patients with intellectual disability and may contribute to reduced restrictions. However, in the present case series, the observed adverse effects included osteoporosis/osteopenia and suppression of male pubertal development during treatment with GnRH agonists. These findings are consistent with the known hypogonadal effects of TLM. More broadly, testosterone‐lowering treatment is associated with a range of potential adverse effects. In addition to reduced bone mineral density and impaired physical maturation, hypogonadism‐related symptoms such as fatigue, anaemia, metabolic changes, weight gain, gynaecomastia and impaired fertility may occur. Depressive symptoms have been reported and require psychiatric monitoring. Steroidal antiandrogens, including MPA and CPA, have been associated with venous thromboembolism and possible cardiovascular risk, and CPA carries a dose‐dependent risk of meningioma. GnRH agonists such as leuprorelin acetate may contribute to long‐term metabolic and skeletal complications. These risks underscore the need for careful individualised risk–benefit assessment, particularly in young patients and those requiring prolonged treatment.

The identification of fewer than five patients in Denmark voluntarily receiving or having received TLM suggests limited access to this intervention. In carefully selected cases, voluntary treatment may contribute to reduced risk of sexual offending and potentially allow fewer environmental restrictions.

However, the use of TLM in individuals with intellectual disability raises important ethical considerations, including assessment of decision‐making capacity, proportionality of intervention, long‐term impact on physical health and fertility, and the risk of implicit coercion in forensic or institutional settings.

While higher‐quality evidence is desirable, classical randomised controlled trials may be difficult to conduct given the rarity of eligible cases, clinical heterogeneity and ethical constraints related to withholding treatment in high‐risk individuals. International collaborative prospective cohort studies and standardised outcome monitoring may represent more feasible approaches to strengthening the evidence base.

## Funding

No funding was received for this manuscript.

## Conflicts of Interest

The authors declare no conflicts of interest.

## Data Availability

The data that support the findings of this study are available upon request from the corresponding author. The data are not publicly available due to privacy or ethical restrictions.
